# CD40 Ligand (CD154) Incorporated into HIV Virions Induces Activation-Induced Cytidine Deaminase (AID) Expression in Human B Lymphocytes

**DOI:** 10.1371/journal.pone.0011448

**Published:** 2010-07-06

**Authors:** Marta Epeldegui, Dharma R. Thapa, Justin De La Cruz, Scott Kitchen, Jerome A. Zack, Otoniel Martínez-Maza

**Affiliations:** 1 Departments of Microbiology, Immunology and Molecular Genetics, David Geffen School of Medicine at University of California Los Angeles, Los Angeles, California, United States of America; 2 Department of Medicine, David Geffen School of Medicine at University of California Los Angeles, Los Angeles, California, United States of America; 3 University of California Los Angeles AIDS Institute, Los Angeles, California, United States of America; 4 Department of Obstetrics and Gynecology, David Geffen School of Medicine at University of California Los Angeles, Los Angeles, California, United States of America; 5 Jonson Comprehensive Cancer Center, Los Angeles, California, United States of America; 6 Department of Epidemiology, School of Public Health, University of California Los Angeles, Los Angeles, California, United States of America; University of Toronto, Canada

## Abstract

Most AIDS-associated non-Hodgkin's lymphoma (AIDS-NHL) arises from errors in immunoglobulin heavy-chain gene (*IgH*) class switch recombination (CSR) or somatic hypermutation (SHM), events that occur in germinal center (GC) B cells and require the activity of activation induced cytidine deaminase (AID). Several oncogenic viruses (EBV, HCV, HPV) can induce AID gene (*AID*) expression, and elevated *AID* expression is seen in circulating lymphocytes prior to AIDS-NHL diagnosis. Here, we report that HIV produced in peripheral blood mononuclear cells (PBMC) induced *AID* expression in normal human B cells. Since HIV produced in PBMC contains host cell CD40 ligand (CD40L) incorporated into the viral membrane, and CD40L is known to induce *AID* expression in human B cells, the role of virion-associated CD40L in HIV-induced *AID* expression was examined. Only viruses expressing functional CD40L were seen to induce *AID* expression; CD40L-negative HIV did not induce *AID* expression. The induction of *AID* expression by CD40L+ HIV was abrogated by addition of blocking anti-CD40L antibody. AID protein was detected in B cells exposed to CD40L+ HIV using intracellular multicolor flow cytometry, with most AID producing B cells expressing the CD71 activation marker on their surface. Therefore, HIV virions that express CD40L induce *AID* expression in B cells, and this induction appears to be due to a direct interaction between CD40L on these viruses and CD40 on B cells. These findings are consistent with a role for HIV in the direct stimulation of B cells, potentially leading to the accumulation of molecular lesions that have the potential to contribute to the development of NHL.

## Introduction

It has been known for some time that HIV infection is associated with chronic B cell hyperactivation [1], [2], [3], [4], and also that levels of several cytokines and immune system molecules associated with B cell activation are elevated prior to the development of AIDS-NHL [5], [6], [7], [8], [9], [10]. The activation of B cells can result in the expression of activation-induced cytidine deaminase (AID), a DNA-mutating enzyme that plays a central role in two DNA-modifying activities normally seen to follow B cell activation: immunoglobulin heavy chain gene (*IgH*) class-switch recombination (CSR) and somatic hypermutation (SHM) [11]. The expression of the AID gene (*AID*) also has been seen to be associated with the mutation and/or translocation of oncogenes associated with NHL; therefore, *AID* expression and activity is thought to play a seminal role in the genesis of B cell NHL [12].

AIDS-associated non-Hodgkin's lymphoma (AIDS-NHL) is a common cancer in HIV infected (HIV+) subjects [13]. In fact, NHL is the most common AIDS-related cancer in HIV+ populations that have access to effective anti-retroviral treatment [14], [15]. While virtually all AIDS-NHL are B cell tumors, these tumors are heterogeneous, representing different types of NHL, which differ mainly in terms of molecular lesions, as well as in infection of tumor cells with oncogenic viruses. AIDS-NHL are thought to arise from: 1) loss of immunoregulatory control of Epstein-Barr Virus (EBV) infection of B cells, and/or 2) chronic immune activation of B cells, associated with immune system dysfunction caused by HIV infection, and resulting in the accumulation of oncogenic molecular lesions [16]. Therefore, both uncontrolled viral infection and B cell activation-associated DNA damage can promote the development of AIDS-NHL.

In recent work, we showed that *AID* is elevated prior to AIDS-NHL diagnosis, in some cases over a period of several years [17]. Infection of B cells by EBV can result in the transformation of such cells, potentially resulting in EBV+ lymphomas in the setting of severe immune deficiency. However, EBV and other viruses, including hepatitis C virus (HCV), also can induce *AID* expression and oncogene mutation, providing another means by which oncogenic viruses could contribute to lymphomagenesis [18] [19], [20]. Little is known about the mechanism(s) involved in the direct induction of *AID* expression by oncogenic viruses.

It has been thought that the contribution of HIV infection to the development of AIDS-NHL is due to indirect effects, such as the loss of T cells responsible for controlling infection with oncogenic viruses, or by the HIV-driven overproduction of B cell stimulatory factors such as cytokines [7] or ferritin [21], rather than mediated via a direct interaction between HIV virions and B cells. However, there is evidence that HIV itself can directly induce polyclonal B cell activation. Schnittman and co-workers reported that HIV could directly induce B cell activation more than twenty years ago [22]. More recently, it has been reported that HIV can directly induce B cell activation, via either gp120:DC-SIGN interactions [23], or via the stimulation of CD40+ B cells by CD40 ligand (CD40L) incorporated into HIV virions [24], or perhaps via interactions with Toll-like receptors (TLR) [25]. Therefore, it appears that direct HIV:B cell interactions can result in the activation of these cells, which has the potential to contribute to the generation of molecular errors that result in the development of B cell lymphoma. This possibility led us to examine directly the potential for HIV to induce *AID* expression in human B cells. It was seen that exposure of B cells to HIV led to *AID* expression, and that this did not require infection of B cells. Additionally, it was seen that HIV virion-associated CD40L (CD154) was responsible for the induction of *AID* expression in human B cells.

## Results

### Exposure of human B cells to HIV results in the induction of *AID* expression

**Figure 1:**
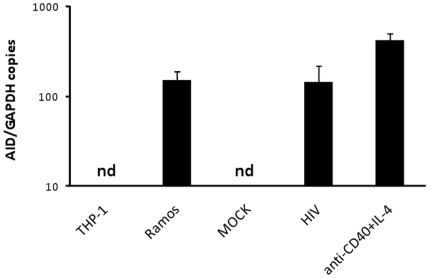
HIV induces AID expression. Taqman RT-PCR was performed used to quantify AID and GAPDH expression in: the monocytic cell line THP-1 (THP-1), Ramos cell line (Ramos), or B cells that were exposed to mock (MOCK) or HIV-containing supernatant (HIV) for 3 days, or treated with anti-CD40 and IL-4 (anti-CD40+IL-4) (positive control) for 3 days. n.d: non-detectable.

B cells were purified from PBMC isolated from healthy donors, exposed to HIV_NL4-3_ for 2 hours, and then kept in culture for 3 days. Following this, *AID* expression was measured by RT-PCR or Taqman RT-PCR. As a positive control for *AID* expression, we used the Ramos cell line, and as a negative control we used the monocytic cell line THP-1, which does not express *AID*. When circulating B cells were exposed to HIV_NL4-3_ virions produced in PBMC, we observed induction of *AID* mRNA expression (Figure 1). In contrast, when B cells were exposed to mock supernatant, produced from uninfected PBMC, *AID* expression was not induced. When B cells were exposed to anti-CD40 and IL-4 as a positive control, *AID* expression was also seen to be induced at high levels. The Taqman RT-PCR assay used in this study was developed specifically to detect only active *AID* and not an AID splice variant (*AIDvar*) that is expressed in unstimulated circulating B cells [17], [20]. We observed that B cells exposed to HIV_NL4-3_ expressed *AID*, at a level approximately 35% of that seen in B cells stimulated with agonistic anti-CD40+IL-4, which was used as a positive control (Figure 1).

There was no change in p24 expression after B cells were exposed to HIV_NL4-3_ (data not shown), making it unlikely that HIV infection of B cells is necessary for *AID* expression, since viral replication did not appear to occur in these cultures.

### NL4-3 and JR-CSF viruses incorporate CD40L

It has been shown by others [24], [26] that CD40L can be incorporated into HIV's viral envelope membrane upon viral budding from infected cells. Since direct HIV infection of B cells did not occur, this appeared unlikely to be responsible for the observed *AID* induction following exposure of B cells to HIV. Given that stimulation of B cells by CD40L is known to potently induce *AID* expression, we considered the possibility that the interaction between HIV virion-associated CD40L and CD40 on B cells was responsible for the induction of *AID* expression seen upon exposure to HIV.

**Figure 2:**
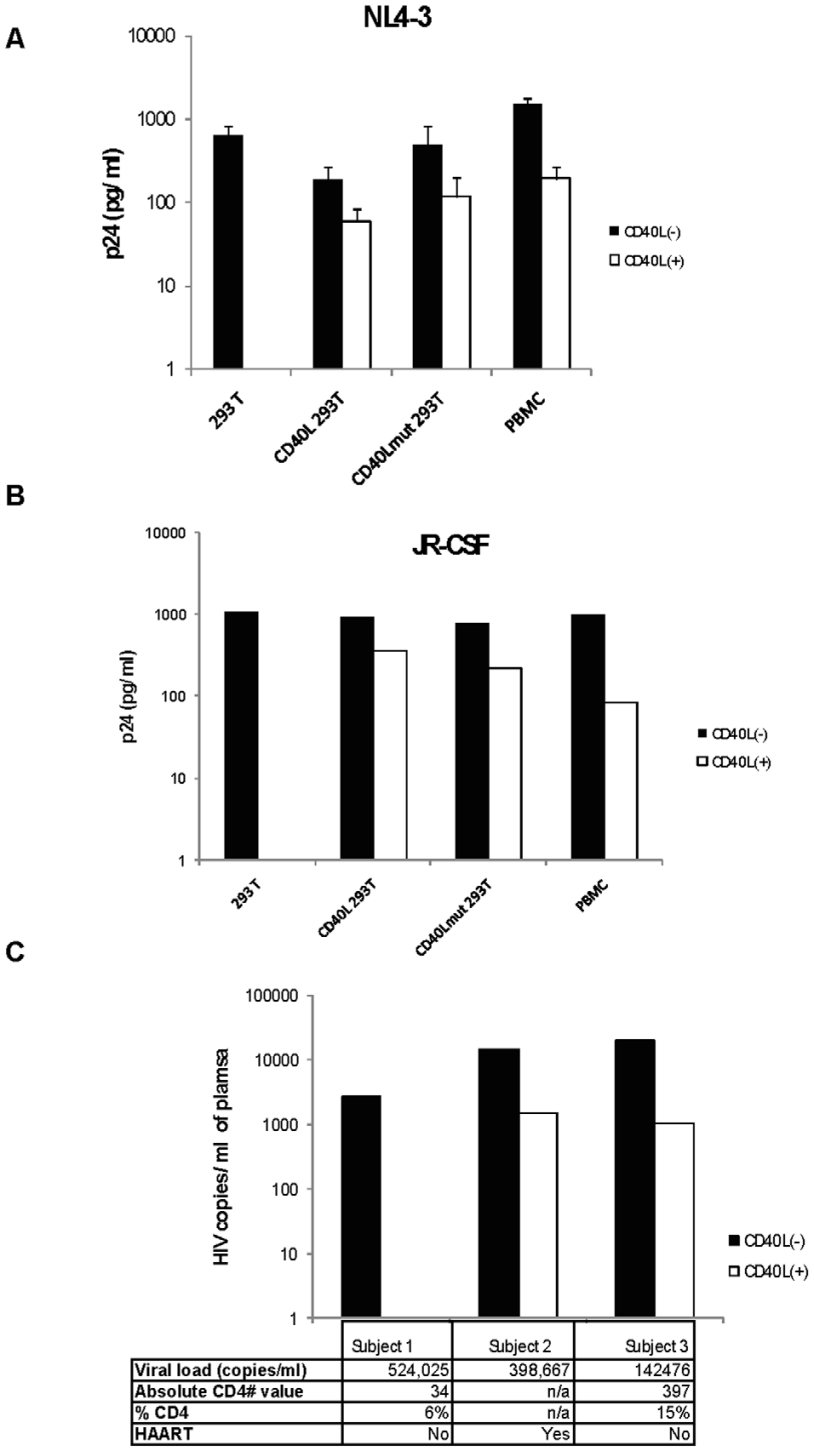
HIV virion-associated CD40L. Viruses (NL4-3 (**A**) or JR-CSF (**B**)) were grown in 293T cells (293T), 293T cells that expressed CD40L (CD40L 293T), 293T cells that expressed non-functional mutant CD40L (CD40Lmut 293T), or PBMC (PBMC), then CD40L expressing viruses were purified using magnetic beads coated with anti-CD40L, followed by assessment of HIV content by quantification of HIV p24 levels by ELISA. Black bars represent virions (NL4-3 or JR-CSF) not bound to beads, which are therefore CD40L-negative virions, (CD40L(−)) and white bars represent virions (NL4-3 or JR-CSF) bound to beads, which are therefore CD40L-positive virions (CD40(+)). **C**) Plasma from HIV-seropositive subjects was incubated with magnetic beads coated with anti-CD40L, followed by magnetic separation. RNA was then purified from the viruses bound to the beads, and were CD40L-positive virions (CD40L(+)), as well as the viruses that did not bind to beads and were CD40L-negative virions (CD40L(−)), followed by Taqman RT-PCR for viral RNA. Viral load, absolute CD4 number, percent CD4 cells (% CD4) and treatment status is listed for each subject, where available (n/a =  not available).

In order to determine if HIV virion-associated CD40L has the capability to induce *AID* expression in circulating B cells, we produced HIV virions that contained CD40L, as well as HIV that was CD40L-negative. To do this, we transfected 293T cell line with *CD40L* or *CD40Lmut* (T147N) plasmids, along with HIV_NL4-3_ or HIV_JR-CSF_ plasmids. T147N is a mutant CD40L isolated from patients with hyper-IgM syndrome; this CD40L mutant is recognized by CD40L antibodies but does not effectively stimulate B cells via interactions with CD40. It was confirmed by flow cytometry that 293T cells did not express membrane CD40L (data not shown). However, when 293T cells were transfected with CD40L or CD40Lmut, surface membrane expression of CD40L was detectable (not shown). In order to confirm that these viruses did, or did not express CD40L or CD40Lmut, we purified the viruses with magnetic beads coated with anti-CD40L, and then measured p24 expression by ELISA. Viruses grown in 293T cells that were co-transfected with, and expressed, CD40L or mutant CD40L, were seen to bind to CD40L antibody-bound beads (Figure 2), confirming that these HIV virions did express CD40L.

We also confirmed the presence of CD40L in virions grown in activated-PBMC (Figure 2a), as it had been previously reported that both NL4-3 and JR-CSF incorporated CD40L when produced in such cells *in vitro* [26]. Additionally, we isolated CD40L-positive HIV from the plasma of HIV+ subjects, confirming that incorporation of CD40L into HIV virions also occurs *in vivo* (Figure 2c).

### NL4-3 and JR-CSF viruses that are CD40L+ induce *AID* expression in B cells

**Figure 3:**
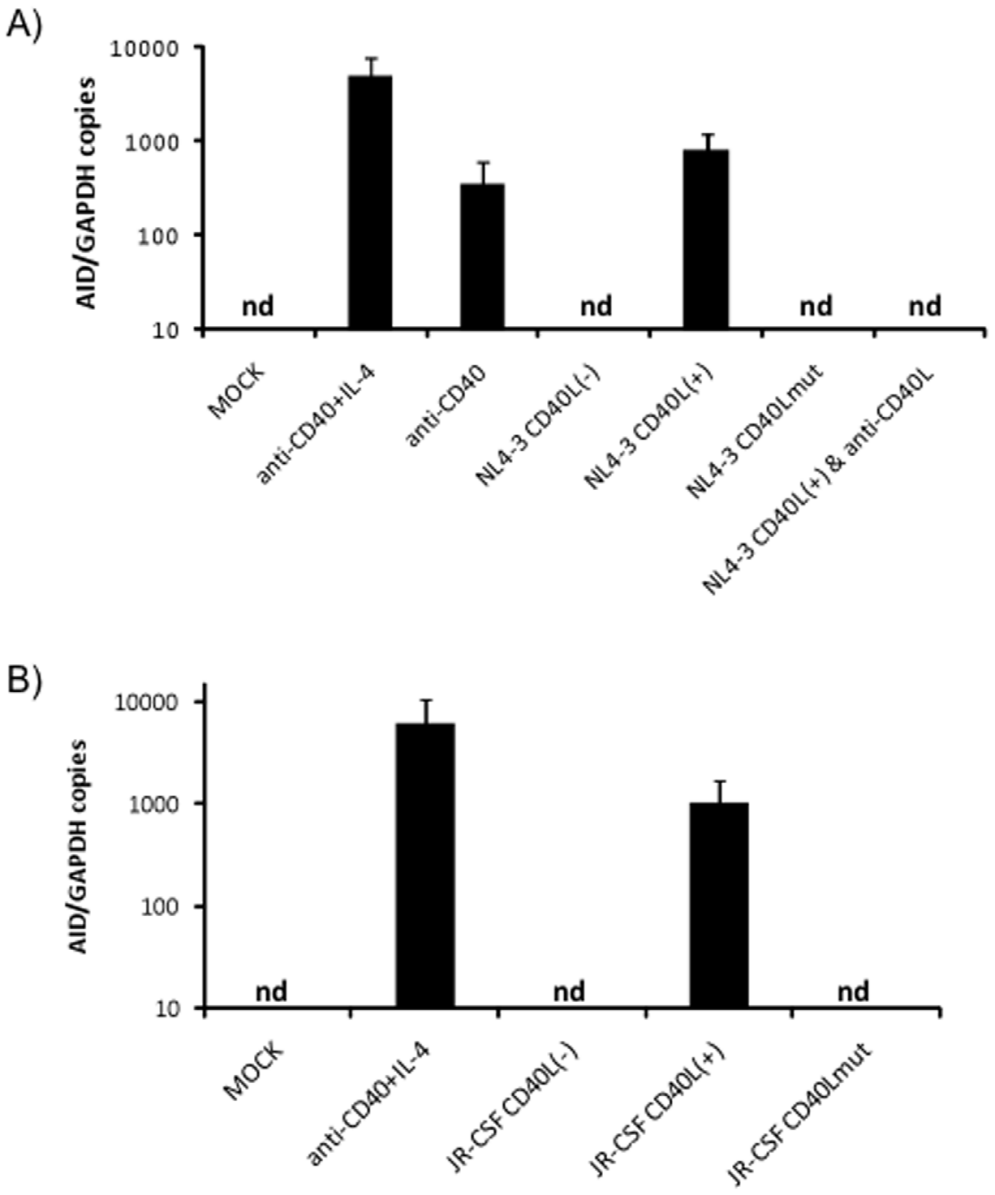
CD40L-expressing HIV, but not CD40L-negative HIV, induced *AID* expression in B cells. **a**) TaqMan RT-PCR was used to asses the expression of *AID* in B cells that were exposed to mock supernatants of 293T cells, which were not transfected (MOCK) (negative control), or exposed to an agonistic anti-CD40 antibody+IL-4 (anti-CD40+IL-4) (positive control), stimulatory anti-CD40 antibody alone (anti-CD40), CD40L-negative HIV-containing supernatants produced by transfecting 293T cells with NL4-3 (NL4-3 CD40L(−)), CD40L-positive HIV-containing supernatants of 293T cells co-transfected with NL4-3 and with CD40L (NL4-3 CD40L(+)) or CD40L mutant (NL4-3 CD40Lmut). B cells were also exposed to these different NL4-3 preparations and cultured for 3 days. B cells exposed to CD40L-positive HIV were also treated with anti-CD40L. **b**) TaqMan RT-PCR was used to asses the expression of *AID* in B cells that were exposed to supernatants of 293T cells that were not transfected with HIV (MOCK), stimulatory anti-CD40 antibody + IL-4 (anti-CD40+IL-4) (positive control), CD40L-negative HIV-containing supernatants produced by transfecting 293T cells with JR-CSF (JR-CSF CD40L(−)), CD40L-positive HIV containing supernatants of 293T cells co-transfected with HIV and with CD40L (JR-CSF CD40L(+)), or CD40L mutant (JR-CSF CD40Lmut). B cells were exposed to these different JR-CSF preparations and cultured for 3 days.

After confirming that HIV produced in 293T cells expresses CD40L upon co-transfection with CD40L (or mutant CD40L), we exposed purified circulating B cells to these different viral preparations. It was seen that the HIV virions (NL4-3 or JR-CSF) produced in 293T cells co-transfected with HIV and CD40L were able to induce *AID* expression in B cells (Figure 3). On the other hand, the virions that expressed either CD40Lmut (which does not stimulate B cells), or did not express CD40L (virions produced in cells that were not co-transfected with CD40L), were not able to induce *AID* expression in B cells, suggesting that HIV is stimulating B cells in a direct manner via virion-associated CD40L interaction. B cells were also exposed to supernatants from 293T cells, or 293T cells that were transfected with either CD40L or CD40L mutant, but not with HIV. None of these preparations induced *AID* expression in B cells.

Addition of a blocking antibody to CD40L resulted in the abrogation of *AID* expression induced by CD40L-positive HIV preparations (Figure 3), confirming that the induction of *AID* expression in B cells is due to the CD40L:CD40 interaction. Treating the B cells with this blocking anti-CD40L antibody, without added virus, did not appear to have any effect on *AID* expression in B cells. It is also interesting to note that both X4 and R5 viruses were seen to have the ability to incorporate CD40L and to induce *AID* expression in B cells (Figure 3).

We wanted to confirm that HIV infection of B cells was not required for B cell activation and induction of *AID* expression, so B cells were exposed to HIV virions containing CD40L, and simultaneously exposed to zidovudine (AZT), to prevent viral replication. In the presence of AZT, *AID* expression was still seen in CD40L+ virion-exposed B cells, confirming that viral replication does not appear to be required for the induction of *AID* expression by these CD40L+ HIV preparations (data not shown).

### CD40L positive HIV virions induce AID protein expression in B cells

**Figure 4:**
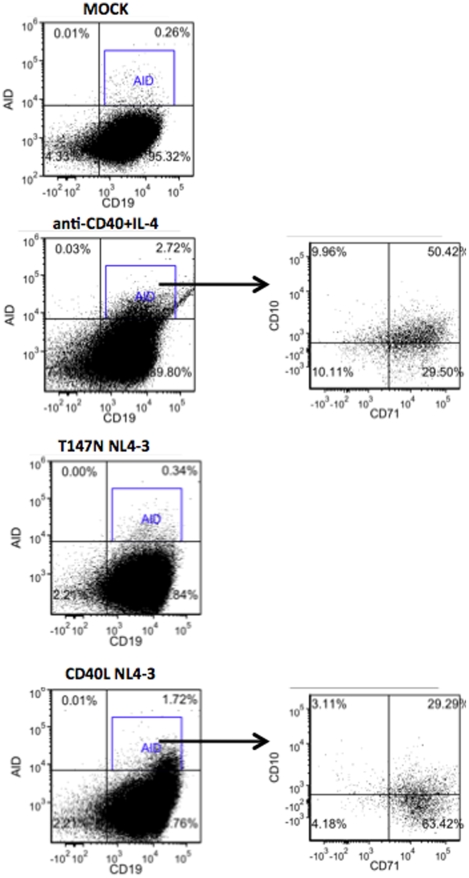
Exposure of B cells to CD40L bearing HIV_NL4-3_ virions resulted in AID protein expression. Flow cytometry for the detection of intracellular AID protein was performed for B cells stimulated with anti-CD40+IL-4, Mock (HIV-negative), T147N (non-functional mutant CD40L) and CD40L-expressing HIV_NL4-3_ supernatants. B cells (CD19+) expressing intracellular AID protein were gated and cell-surface expression of CD10 and CD71 was assessed on these AID+ cells. The results of a representative experiment are shown in this figure.

We wanted to determine if the induction of AID gene expression following exposure of B cells to CD40L+ HIV resulted not only in elevated levels of AID mRNA, but also in elevated levels of AID protein. For this purpose we measured AID protein expression by intracellular multi-color flow cytometry in B cells that had been exposed to CD40L positive or CD40Lmut (T147N) NL4-3 HIV preparations, grown in CD40L(+) or T147N(+) 293T cells. AID protein was seen in B cells exposed to CD40L-positive HIV_NL4-3_, as well as in B cells stimulated with anti-CD40+IL-4: 2.11±0.55 (SD)% of CD19+ cells exposed to CD40L+ HIV_NL4-3_ for three days expressed intracellular AID, while 0.25±0.13% of B cells exposed to CD40Lmut+ HIV_NL4-3_ and 0.17±0.13% of B cells exposed to mock supernatants were AID+ (P<0.02, t test comparing CD40L+ HIV_NL4-3_ to mock supernatant negative control). There was no significant increase in AID protein expression detected in cells exposed to mock preparations (virus negative) or to HIV_NL4-3_ incorporating mutant CD40L (Figure 4). To determine if these AID positive B cells displayed other markers of B cell activation, multi-color flow cytometry was performed for intracellular AID, and cell surface CD19, CD10 and CD71. CD71, the transferrin receptor, is an activation marker for human B cells. It has been seen by others that the majority of AID-positive GC B cells also co-express CD71 [27]. Additionally, in a prior study we found that elevated levels of CD71+ and CD10+ B cells were seen in the circulation of persons with HIV infection and AIDS [3]. We observed exposure of human B cells to CD40L-positive HIV (or to anti-CD40+IL-4) led to a significant increase in CD71 expression on these cells: 26±2.8% of CD19+ cells exposed to CD40L+ HIV_NL4-3_ expressed CD71 on their surface, while 2.2±1.1% of B cells exposed to CD40Lmut HIV_NL4-3_ and 1.7±1.3% of B cells exposed to mock supernatants were CD71+ (P<0.004, t test comparing CD40L+ HIV_NL4-3_ to mock supernatant negative control). The majority of B cells that expressed AID following exposure to CD40L-positive HIV also expressed CD71: 94±4.4% of AID+ B cells exposed to CD40L-positive HIV_NL4-3_ supernatants co-expressed CD71. Additionally, 19±6.8% of the AID+ B cells seen following exposure to CD40L-positive HIV also co-expressed CD10, a marker for immature and GC B cells [3], [27], [28]. Together, this suggests that the AID expression induced in B cells following exposure to CD40L-positive HIV occurs in cells that have been induced to express an activated/GC phenotype.

### CD40L positive HIV virions induce cytokine secretion by virus-exposed B cells

Cytokine expression was also measured in the supernatants of B cells that were exposed to CD40L+ virions, as well as positive and negative control stimuli, in two independent experiments. IL-2, IL-10, GM-CSF, IL-6 and IL-8 were all elevated in B cells exposed to CD40L+ HIV preparations when compared to controls (Figure S1).

## Discussion

Defects in B cell immunity and chronic B cell activation have been recognized as features of HIV infection and AIDS for some time [1], [2], [3], [4]. However, the role of direct interactions between HIV and B cells in this process has been less clear. Early reports indicated that HIV could directly stimulate B cell activation [22], but for the most part, B cell activation in the setting of HIV infection has been thought to be due to HIV disease-associated immune dysfunction, such as loss of immunoregulatory T cell control of EBV infected B cells, response to HIV antigens [29], superantigen stimulation of B cells [30], and/or the overproduction of inflammatory and B cell-stimulatory cytokines [31], [32], [33] [34]. However, more recently, some reports have indicated that HIV may be able to induce B cell activation directly. For instance, a recent report by He and co-workers [23] concluded that HIV gp120 was interacting with receptors on B cells, including DC-SIGN, leading to B cell activation (class switch recombination, *AID* expression and interleukin secretion). Other reports, by Martin and co-workers [35] [26], concluded that CD40L, an immune stimulatory molecule expressed by activated T cells, is incorporated into HIV virions and can stimulate B cells via CD40L:CD40 interactions. However, the effect of virion-incorporated CD40L on *AID* expression was not examined in that study. These recent reports defined molecular interactions that might play a role in the direct activation of B cells by HIV.

Here we confirm that HIV does indeed appear to interact directly with B cells through the interaction of CD40L with CD40, and report that this interaction results in the induction of AID gene and protein expression in resting human B cells, isolated from the PBMC of HIV-negative donors. Elevated cytokine secretion, as well as expression of CD71, a marker of cellular activation, also was seen in B cells stimulated by exposure to CD40L-containing HIV, with most AID expressing cells also co-expressing CD71. Additionally, many AID expressing cells were CD10 positive. This indicates that these HIV-stimulated AID expressing B cells have an activated/GC phenotype.

These findings are notable, as AID is a DNA-modifying enzyme that is believed to play a central role in the development of B cell NHL. Since AID is a central effector in both *IgH* CSR and SHM [11], [12], and errors (oncogene translocation and/or mutation) in these processes lead to the development of NHL, the induction of *AID* expression has the potential to play a seminal role in the genesis of these cancers. We also found that plasma HIV from some HIV+ subjects was CD40L-positive, indicating that the incorporation of CD40L into virions also occurs *in vivo*. Because of this, the B cell activation observed in these studies has the potential to also occur *in vivo*. Further studies are needed to show if HIV-associated host cell immune stimulatory molecules are involved in the induction of *AID* expression *in vivo*.

The observation that HIV virions can interact directly with resting human B cells to induce *AID* expression, without the requirement for infection, and via the interaction of virion-associated CD40L with CD40 on B cells, provides a direct means by which HIV may contribute to the development of AIDS-NHL. In a recent study, we noted that elevated levels of *AID* expression were seen in those HIV+ subjects who went on to develop NHL, with the highest levels seen in those who developed Burkitt's lymphoma [17], a form of B cell NHL that is characterized by the *MYC:Ig* chromosomal translocation, a result of AID activity [35], [36].

Our results also suggest that direct interactions between HIV-encoded molecules, such as env protein, and receptors on human B cells, are not responsible for HIV-mediated *AID* expression, as CD40L-negative HIV preparations were not seen to induce *AID* expression in agreement with the studies carried by Martin et al [24]. This is in contrast to the results of studies by He, et al [23], in which it was seen that HIV env had B cell-stimulatory effects. These different results may be due, in part, to differences in the source of HIV env, as He, *et al* used soluble envelope protein, while we exposed B cells to infectious HIV preparations. Also, the B cell preparations utilized in that study were different from those used in the current study. It is important to note that the results presented here do not exclude the potential role of other immune stimulatory molecules of host origin that are incorporated into HIV virions in the induction of *AID* expression by B cells, as it is certainly possible that virion-associated host cell-encoded molecules other than CD40L may be contributing to B cell activation.

Interestingly, other viruses (EBV, HCV) and microbes (*H. pylorii*) are known to be able to induce *AID* expression, and are associated with the development of B cell lymphoma [18], [19], [20], [37]. In this study, we found that exposure of B cells to HIV resulted in marked *AID* expression by three days post-exposure, while exposure to EBV required ten days for optimal *AID* expression [20]. This difference in the kinetics of induction of *AID* expression by these two viruses may be due to EBV having to infect B cells and express viral proteins (i.e., LMP1) in order to induce *AID* expression, while HIV may induce *AID* expression more quickly, because it does so by direct CD40L:CD40 stimulation, which does not require viral infection.

Finally, it is important to note that chronic antigenic and/or cytokine-mediated stimulation of B cells, in the setting of HIV infection-associated immune dysfunction, also would be expected to result in *AID* expression. Therefore, the interaction of CD40L+ HIV virions with B cells is clearly not the only mechanism that has the potential to induce aberrant *AID* expression, and contribute, potentially, to the accumulation of molecular lesions that lead to NHL. Clearly, further study is needed to determine the relative contribution of virion-associated CD40L to the induction of B cell activation and *AID* expression in HIV infected persons, as well as to determine if other similar immune activation molecules incorporated into HIV virions are active in inducing B cell activation and AID gene expression and activity.

## Materials and Methods

### CD40L and T147N HIV

pCD40L (CD40L) and pT147N (CD40L mutant) was kindly provided by R. Kornbluth (University of California San Diego). pNL4-3 or pJR-CSF HIV molecular clones were obtained through the AIDS Repository Reagent Program (Germantown, MD). 293T human embryonic kidney cells (ATCC CRL-11268) were transfected with these HIV molecular clones [38]; these cells were plated at 2×10^5^cells/well in 0.5 ml of RPMI with 1% glutamine in a 24 well plate. For each well, 1 µg of pCD40L or pT147N and/or 0.5 µg of pNL4-3 or pJR-CSF was diluted in 50 µl of RPMI (for co-transfections the total DNA was 1.5 µg) and 1.2 µl of lipofectamine in 50 µl of RPMI. Lipofectamine-DNA complexes were incubated for 20 min at room temperature, added to wells (100 µl per well) and incubated for 3 days at 37°C and 5% CO_2_. Supernatants were collected after 3 days.

### RNA isolation, Taqman RT-PCR

RNA isolation and PCR for *AID* were performed as previously described [20]. Real time PCR measurements were done in duplicate for the samples and in triplicate for the standard curve. *AID* mRNA levels detected by quantitative TaqMan RT-PCR were normalized for total RNA content by dividing the *AID* copy number by the *GAPDH* copy number [20]. This *AID/GAPDH* ratio was then multiplied by 10,000 to provide a relative numerical index of *AID* expression that is adjusted for RNA content [(*AID/GAPDH*)×10,0000]. *AID* expression was considered detectable when the numerical index was >2.0.

### B cell isolation from PBMC

Peripheral blood mononuclear cells (PBMC), from healthy anonymous donors, were obtained from the UCLA AIDS Institute Virology Core laboratory. PBMC were counted and resuspended at 25×10^6^ cells/ml in cold PBS containing 0.1% BSA and 2 mM EDTA. 25 µl of CD19 immunobeads (Dynal Biotech, Invitrogen) was added per 25×10^6^ cells and incubated at 2-8°C for 20 minutes on a rotator. CD19+ cells (B cells) were isolated using a magnet, and washed 2× with PBS containing 0.1% BSA and 2 mM EDTA. Beads were eliminated by adding 100 µl of Detachabead (DAB, Dynal Biotech, Invitrogen) per 1×10^6^cells in culture medium, while incubating for 40 minutes on a rotator. Following this, detached cells were washed two-three times with culture medium, after which cells were pelleted.

### Exposure of B cells to HIV preparations

B cells were incubated with different HIV preparations. 6×10^6^ B cells were exposed to 1 ml of HIV supernatant (100 ng of p24) or Mock supernatant, which are supernatants collected from same uninfected cell lines or PBMC where the virus was grown, for 3 hours. 2×10^6^ B cells were exposed with HIV expressing CD40L, T147N mutant or not expressing CD40L at a concentration of 100 ng of p24 for 2 hours. Cells were then washed with culture media and were incubated for 3 days at 1×10^6^ cells/ml in a 24 cell well plate at 37°C, 5% CO_2_. In order to inhibit viral replication, azidothymidine (AZT) was added at a concentration of 10 µM at the time of HIV exposure, and incubated with HIV for 2 hours, then cells were washed and cultured for 3 days. To block CD40:CD40L interaction, we added blocking anti-CD40L (Ancell, Minnesota) at 0.1 µg/ml at the time of HIV exposures, then cells were washed and incubated for 3 days.

### Isolation of CD40L positive viruses

CD40L+ viruses were isolated from virus preparations by affinity binding, using beads coated with anti-CD40L antibodies. Briefly, this was done by washing sheep anti-mouse IgG Dynal magnetic beads (Dynal Biotech, Invitrogen) with at least 1 ml PBS/0.1% BSA, resuspending these to the original volume. 25 µl of beads were used per 1 ng of p24 in the viral preparations (4 ng of p24 per viral preparation). 1.5 µg of anti-CD40L (3 µl of 0.5 µg/µl antibody) (BD Pharmingen, San Jose, CA) was added per 25 µl of beads. Washed beads were incubated with anti-CD40L for 30 min at 4°C. CD40L antibody-coated were then washed with at least 1 ml PBS/0.1% BSA, and resuspended to their original volume. Viral preparations containing 4 ng of p24 were brought to a volume of 1 ml with PBS/0.1% BSA, added to the beads, and incubated for 20–30 min at 4°C with tilting. Beads bound to CD40L+ virus were then washed 2 times by adding 1 ml of PBS/0.1% BSA, and put into contact with a magnet for 2 minutes. Both, the bead-bound and unbound viruses were disrupted by adding Triton X (final concentration 0.5%), and assayed for p24 content by ELISA (Perkin Elmer), to quantify the amount of CD40L+ and CD40L-negative HIV in these preparations.

1 ml of plasma for each subject was incubated with CD40L labeled beads as described above, then virus bound to beads were resuspended in RLT buffer (Qiagen) and RNA extraction was performed as described in [20]. Virions in plasma were isolated by diluting 1 ml of plasma to 5 ml of PBS, then centrifuged for 90 minutes at 40,000 rpm, then pellet was resuspended in RLT buffer (Qiagen) and RNA extraction was performed as described in [20]. 5 µl of RNA extracts were used for HIV RNA Taqman RT-PCR. [39]

### Measurement of AID protein by flow cytometry

Briefly, 1-2×10^6^ cells were washed with 3 ml of cold PBS, cells were then fixed by adding formaldehyde to a final concentration of 1% and incubating 4°C for 30 minutes. Cells were then washed with PBS/1%BSA by centrifugation at 400 g for 5 minutes. Cells were then permeabilized by adding 1 ml of 0.2% Tween/PBS for 15 minutes at 37°C. After this, cells were washed with 3 ml of PBS/1% BSA, centrifuged, then resuspended in 50 µl of human AB serum and antibodies were then added: 10 µl of 1∶100 dilution of the rat-anti AID primary antibody (Cell Signaling) or rat IgG (final concentration of 1.56 µg/ml) to the assay tubes and incubated for 20 minutes at 4°C. Cells were then washed by adding 3 ml PBS/1% BSA, followed by centrifugation. Cells were resuspended in 50 µl of Human AB serum and secondary antibody goat anti-rat Alexa Fluor® 488 (Invitrogen) was added at a 1∶100 dilution, then incubated for 30 minutes at 4°C. Cells were washed again with 3 ml PBS/5% BSA. Then, cells were resuspended in 50 µl of Mouse IgG (Caltag) diluted 1∶15 and incubated for 10 min at 4°C. Subsequently, labeled antibodies were added: 10 µl of CD10-PE-Cy7 (BD), 10 µl of CD71-PE (BD Pharmingen) and 10 µl CD19-Cy7-PE (BD Pharmingen), incubated for 20 minutes at 4°C, washed with 3 ml of PBS/1%BSA and resuspended in 300 µl of PBS/1%BSA followed by analysis by flow cytometry.

### Plasma specimens

For the assessment of CD40L expression on virions from the plasma of HIV-infected subjects, archival plasma specimens were utilized. These specimens were collected from HIV+ and HIV-negative subjects who were in the UCLA Center of the Multicenter AIDS Cohort Study (MACS) or were patients in UCLA clinics.

### Quantification of cytokine levels by multiplexed immunometric assay

Levels of human cytokines in the culture supernatants of HIV-exposed B cells were quantified using a multiplexed (Luminex platform) immunometric assay (MILLIPLEX xMAP High Sensitivity Human Cytokine Panel, Millipore). This assay can simultaneously measure levels of the following human cytokines: GM-CSF, IFNγ, IL-1β, IL-2, IL-4, IL-5, IL-6, IL-7, IL-8, IL-10, IL-12 (p70), IL-13 and TNFα. Supernatant cytokine levels were assessed in two independent experiments.

## Supporting Information

Figure S1 Exposure of B cells to CD40L bearing HIVNL4-3 virions resulted in increased cytokine expression. Multiplexed immunometric assays for the detection of cytokines (Milliplex High Sensitivity Human Cytokine Panel, Millipore) were performed on supernantants of B cells stimulated with anti-CD40L+IL-4, Mock (HIV-negative), T147N (non-functional mutant of CD40L) and CD40L-expressing HIVNL4-3. The results shown represent mean cytokine levels, and standard error of the mean. The asterisk indicates that supernatant levels of secreted IL-4 were not reliably assessed, as exogenous IL-4 had been added to those cultures.
(1.56 MB TIF)
