# Supplementary figures and images for: CD40 Ligand (CD154) Incorporated into HIV Virions Induces Activation-Induced Cytidine Deaminase (AID) Expression in Human B Lymphocytes
Source: PLoS One. 2010 Jul 6;5(7):e11448. doi: 10.1371/journal.pone.0011448 (PMC2897846; doi:10.1371/journal.pone.0011448)

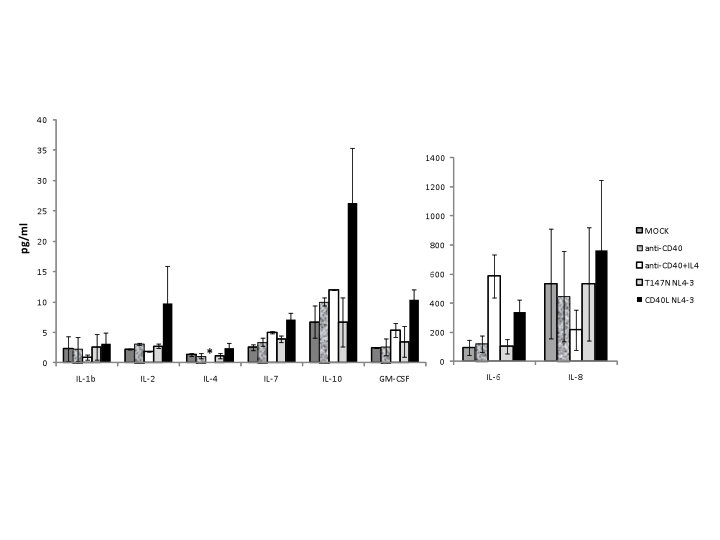

Supplement: Figure S1 — Exposure of B cells to CD40L bearing HIVNL4-3 virions resulted in increased cytokine expression. Multiplexed immunometric assays for the detection of cytokines (Milliplex High Sensitivity Human Cytokine Panel, Millipore) were performed on supernantants of B cells stimulated with anti-CD40L+IL-4, Mock (HIV-negative), T147N (non-functional mutant of CD40L) and CD40L-expressing HIVNL4-3. The results shown represent mean cytokine levels, and standard error of the mean. The asterisk indicates that supernatant levels of secreted IL-4 were not reliably assessed, as exogenous IL-4 had been added to those cultures. (1.56 MB TIF) [file pone.0011448.s001.tif]
